# When two fields collide: Identifying “super-recognisers” for neuropsychological and forensic face recognition research

**DOI:** 10.1177/17470218211027695

**Published:** 2021-06-23

**Authors:** Sarah Bate, Emma Portch, Natalie Mestry

**Affiliations:** Department of Psychology, Faculty of Science & Technology, Bournemouth University, Poole, UK

**Keywords:** Super-recognisers, face recognition, face perception, individual differences, psychometrics

## Abstract

In the last decade, a novel individual differences approach has emerged across the face recognition literature. While the field has long been concerned with prosopagnosia (the inability to recognise facial identity), it has more recently become clear that there are vast differences in face recognition ability within the typical population. “Super-recognisers” are those individuals purported to reside at the very top of this spectrum. On one hand, these people are of interest to cognitive neuropsychologists who are motivated to explore the commonality of the face recognition continuum, whereas on the other hand, researchers from the forensic face matching field evaluate the implementation of super-recognisers into real-world police and security settings. These two rather different approaches have led to discrepancies in the definition of super-recognisers, and perhaps more fundamentally, the approach to identifying them, resulting in a lack of consistency that prohibits theoretical progress. Here, we review the protocols used in published work to identify super-recognisers, and propose a common definition and screening recommendations that can be adhered to across fields.

The term “super-recognisers” was coined in a landmark paper by Russell and colleagues (2009), describing four individuals who believed they were “exceptionally good at recognising faces” (p. 252). These individuals reported (a) situations where they had correctly recognised near-strangers who they had not seen for many years and who had undergone major changes in appearance (e.g., via ageing or radical changes in hairstyle), (b) the ability to recognise non-famous actors across minor roles in television shows and advertisements, and (c) the need to moderate their social behaviour to avoid alarming people who do not return their recognition. Since the publication of Russell et al.’s paper, 24^1^ further peer-reviewed empirical investigations (see Supplementary Material [SM1]) have considered super-recognition across the fields of cognitive neuropsychology, experimental psychology, and applied forensic psychology, and a recent special issue of the *British Journal of Psychology* debated real-world implementation of these individuals (Bate, Portch, et al., 2019; Devue, 2019; Moreton et al., 2019; Ramon et al., 2019; Robertson & Bindemann, 2019; Young & Noyes, 2019). Although the three characteristics offered by Russell and colleagues have been noted in occasional further reports (e.g., Noyes et al., 2017), the prevailing, rather informal concept, particularly in the popular press, is simply that super-recognisers “never forget a face.” In essence, this definition is supported by many theoretical investigations where tests of unfamiliar face memory are used to identify super-recognisers (see below), although admittedly no test probes longer-term face memory. However, both the definition and dominant screening protocols are at odds with the forensic face matching literature and real-world application of super-recognisers, where the focus is primarily on forensic facial image comparison—a task that places minimal demands on memory.

This conflict between super-recogniser screening protocols and outcome measures makes the absence of a common scientific definition of the phenomenon particularly concerning. Furthermore, as varying (and sometimes very basic; see below) protocols are used to objectively identify super-recognisers, cross-comparison between studies is often impossible, and there may be substantial variation in the skills of those professed to meet inclusion criteria. Ultimately, both factors may prohibit theoretical progress, but particularly so if meaningful inferences are erroneously drawn from individuals who possess skills that are only “above average,” rather than those who are genuinely drawn from the top few of the population. Given the growing interest in super recognition, across academic fields and end-users, standardisation of terminology and screening protocols is certainly timely, if not urgent. Here, we offer a synthesis of the current state of the art, suggesting a definition and set of inclusion criteria that could reasonably be applied across relevant fields, particularly in the advent of widespread online screening.

## Why study super-recognisers?

Any attempt to offer a definition of super-recognition is necessarily grounded in the motivations for studying the phenomenon. The initial theoretical drive for the study of super-recognisers originated in the cognitive neuropsychological literature, where an individual differences approach to the opposing end of the face recognition spectrum has been active for well over a century (e.g., Jackson, 1876; Wigan, 1844). Here, rare cases of facial identity recognition deficits in the context of acquired prosopagnosia (difficulties that present following neurological illness or injury, typically affecting occipitotemporal areas, e.g., Barton, 2008; De Renzi et al., 1994) have long been used to inform our understanding of the structure and functioning of the typical face recognition system (e.g., Bruce & Young, 1986). In recent decades, a developmental form of the same condition has been reported (e.g., De Haan & Campbell, 1991; Duchaine, 2000; McConachie, 1976) that is more common than its acquired counterpart (Bennetts, Murray, et al., 2017; Bowles et al., 2009), occurring in the absence of neurological injury or other visual, cognitive or emotional dysfunction (Duchaine et al., 2007). However, given anecdotal and objective variation in the severity of face recognition difficulties in developmental prosopagnosia (Adams et al., 2020; Bate, Bennetts, Gregory, et al., 2019; Murray et al., 2018), coupled with apparently broad individual differences in face recognition abilities within the typical population (Wilmer, 2017), it remains unknown whether developmental difficulties truly represent a distinct pathology akin to the acquired form of prosopagnosia (Barton & Corrow, 2016; Bate & Tree, 2017). That is, it is unclear whether a small number of people are impaired relative to the majority of the population, or whether there are simply wide differences in the functioning of people’s face recognition systems, such that those with particularly poor skills reside at the tail end of a much broader distribution of face recognition ability.

Russell and colleagues (2009) offered an innovative means of advancing this debate, reasoning that evidence for the latter explanation could be found if the opposite tail of the spectrum also exists—that is, if some people are as good at face recognition as those with developmental prosopagnosia are bad. They offered support for this viewpoint by presenting the first super-recognisers: four individuals who scored approximately 2 SDs from the control mean (the same criterion that is applied to the identification of prosopagnosia) on multiple tests that are equivalent in process to those used in prosopagnosia diagnosis, albeit with necessary amendments to their calibration (see Tables 1 and 2). Later theoretical investigations into super-recognition have attempted to advance this debate, comparing the process-ing strategies used by super-recognisers to people with developmental prosopagnosia (Bobak et al., 2017; Tardif et al., 2019) or those from the typical population (Bate, Bennetts, et al., 2020; Bate, Bennetts, Hasshim, et al., 2019; Bobak, Bennetts, et al., 2016).

**Table 1.** Face memory tests that have been used to identify super-recognisers in at least two empirical papers.

Task	Reliability	Frequency used (% of studies)		
		Applied (*N* = 13)	Theoretical (*N* = 12)	Overall (*N* = 25)
CFMT+	α = .89^a^	8/13 (61.54)	12/12 (100.00)	20/25 (80.00)
MMT	α = .89^a^	1/13 (7.69)	2/12 (16.67)	3/25 (12.00)
BTWF	Not available	0/13 (0.00)	2/12 (16.67)	2/25 (8.00)
AFRT	Not available	1/13 (7.69)	1/12 (8.33)	2/25 (8.00)

CFMT+: Cambridge Face Memory Test—Long Form (Russell et al., 2009); MMT: Models Memory Test (Bate et al., 2018); BTWF: Before They Were Famous test (Russell et al., 2009); AFRT: Adult Face Recognition Test (Belanova et al., 2018).

Reliability and frequency of use are reported for each test.

a Reliability estimates for these tests are not available in the published literature and were calculated for this article from the 200 self-referred super-recognisers published in Bate et al. (2018).

**Table 2.** Face perception tests that have been used to identify super-recognisers in at least two empirical papers.

Task	Reliability	Frequency used (% of known studies)		
		Applied (*N* = 13)	Theoretical (*N* = 12)	Overall (*N* = 25)
CFPT	α = .53^a^ to .74^b^	0/13 (0.00)	3/12 (25.00)	3/25 (12.00)
GFMT	*R* = .81^c^	4/13 (30.77)	1/12 (8.33)	5/25 (20.00)
PMT	α = .74 to .79^d^	3/13 (23.08)	0/12 (0.00)	3/25 (12.00)

CFPT: Cambridge Face Perception Test (Duchaine et al., 2007); GFMT: Glasgow Face Matching Test (Burton et al., 2010); PMT: Pairs Matching Test (Bate et al., 2018).

Reliability and frequency of use are reported for each test.

a Herzmann et al. (2008).

b Bowles et al. (2009).

c Burton et al. (2010).

d Bate, Bennetts, Hashim et al. (2019).

One line of enquiry examines whether face *perception* skills (in addition to face memory) are facilitated in super-recognisers. The analogous question (i.e., whether face perception skills are impaired in developmental prosopagnosia) is viewed as important in deeming the pathology of the condition, given two broad subtypes of acquired prosopagnosia have historically been reported (those with apperceptive prosopagnosia have an early impairment that affects both face perception and face memory, whereas those with associative or mnemonic prosopagnosia only have difficulties with the latter; De Renzi et al., 1991). It is currently unclear whether the same pattern holds in developmental prosopagnosia, with some evidence supporting the existence of the same two broad subtypes (e.g., Bate, Bennetts, Gregory, et al., 2019; Bate, Bennetts, Tree, et al., 2019; Ulrich et al., 2017), whereas other work suggests that perceptual impairments are much more widespread (Biotti et al., 2019). Currently, there is evidence to support an even wider range of presentations in super recognition, where individuals have been reported with facilitations restricted to only face memory or, on occasion, face perception (Bate et al., 2018; Bate, Frowd, et al., 2019), although most appear to be proficient at both (Bate, Frowd, et al., 2019). Fundamentally, it remains unclear whether these findings genuinely indicate different phenotypes of super recognition, or simply result from the poor psychometric properties of existing screening tests (Young & Noyes, 2019).

This issue is particularly important for the more applied line of forensic face recognition research that has developed in tandem with the cognitive neuropsychological literature. Here, there has been a rapid increase in interest in super-recognisers that has been paralleled, if not preceded (Ramon et al., 2019), by the mobilisation of super-recognisers in real-world policing and security settings, typically for perceptual tasks such as forensic facial image matching or person-to-identification document comparison. While laboratory implementations of the tasks have been used for some years within the forensic face matching literature, the field has been particularly slow to acknowledge the importance of psychometric standards in its relatively newfound individual differences approach. In fact, attempts to assess individual variation in performance have often adopted tasks that were originally created for group-means comparisons (Bindemann et al., 2012; Fysh et al., 2020; Russ et al., 2018), rather than carefully developed, psychometric-standard, normalised tests, with appropriate reliability, validity and sensitivity (Bate, Mestry et al., 2020).

## Relevant issues in psychometric assessment

In psychometrics, issues of test reliability are paramount (Mollon et al., 2017), because any performance indicator intertwines the person’s actual ability with a variety of extraneous factors (e.g., response bias or fluctuating levels of motivation; Young & Noyes, 2019). Calculating the reliability of a particular task is an important means of addressing this issue, both by assessing how consistently a participant performs across trials, and by charting disparities across different performers.

Furthermore, the calibration of the task needs to be appropriate. Because super-recogniser screening tests should have sufficient sensitivity to distinguish between top-performers, control mean performance needs to be sufficiently distanced from ceiling. The typical cut-off used in neuropsychology to detect significantly atypical performance is calculated as the value that is two standard deviations from the control mean (Schinka et al., 2010). For super-recogniser screening, it would be useful for ceiling to exceed 3 SDs from the control mean, to tap further variability within top-end performance. This requirement excludes many tasks that are used for the detection of prosopagnosia, and even some that are used to tap individual differences within the typical population (Fysh et al., 2020; Noyes et al., 2018; Stacchi et al., 2020).

Given task calibration is heavily dependent on the performance of typical perceivers, norming data must be sufficient and appropriate. A large sample size is fundamental, particularly when administering tasks online via participant recruitment platforms that tend to result in large proportions of data loss (Zhou et al., 2016). Given the increased trend in online testing, it is also necessary to use norms that have been collected through the same mode of administration, and under equivalent cognitive load (e.g., performance can differ when participants complete one task in isolation, compared to when they complete the same task among others in the same testing session, even when administered in the same order).

While there is evidence to suggest small gender effects in face recognition performance (e.g., Herlitz & Lovén, 2013; Lovén et al., 2011), these are not substantial and separate norms are seldom applied. However, there is more convincing evidence for considerable age effects on face recognition performance, where findings suggest that ability peaks in the early 30s, and substantial decline begins at the age of 50 years (Germine et al., 2011). Furthermore, ethnicity effects are notorious in face recognition (Meissner & Brigham, 2001), even affecting super-recognisers (Bate, Bennetts, Hasshim, et al., 2019; Robertson et al., 2020). We therefore suggest that potential super-recognisers are compared to a large number (*N* > 100; Garrido et al., 2018) of age- and ethnicity-matched controls, tested via the same online platform as super-recognisers, in a comparable environment.

While these considerations go some way to creating an adequate control sample, one issue that has received very little attention to date is that of participant motivation. Typically, control participants have very little incentive to perform a task to the best of their ability—they will receive the advertised course credit or financial incentive irrespective of their score on the test. For prospective super-recognisers, however, the situation can be very different, and participants often volunteer for screening in the belief that there are higher stakes on offer. While some self-refer for screening out of mere interest or with the wish to assist scientific progress, our laboratory has been contacted by hundreds of people seeking positions of employment or societal status if they reach inclusion criteria. Such misconceptions result from high-profile media coverage of a small number of individuals who have gained super-recogniser employment in the private sector, or officers who were already employed by the police prior to discovery of their skill (e.g., Moshakis, 2018). This issue prompts two concerns. First, it is often the case that individuals who self-refer for super-recogniser screening have already participated in screening with other laboratories, or have previously accessed publicly available tests in preparation for formal screening. This makes issues of test–retest reliability and practice effects particularly important. Second, it is unclear whether existing control groups offer appropriate norms: given the difference in motivation, control norms may be artificially low and offer a liberal cut-off for super recognition.

## Super-recogniser screening tests

Having outlined psychometric issues that are relevant to the development of appropriately calibrated screening tests, we now turn to the available tasks themselves. As stated above, a small variety of tests have been adopted by different laboratories when screening for super-recognition. Here, we primarily sub-divide these tests into two categories: those that measure face memory (see Table 1), and those that assess face perception (see Table 2). We include only tests that have been used in more than one empirical peer-reviewed paper (excluding pre-prints and conference proceedings) for the specific purpose of super-recogniser screening (that is, deeming inclusion criteria for specific studies), and exclude those that have been used as experimental tasks once super-recogniser status has been confirmed.

Tables 1 and 2 evaluate each test in terms of its known psychometric properties, focusing on task reliability, and consider the frequency that each test has been used in super-recogniser screening, splitting this tally according to sub-discipline and aim (i.e., theoretical versus applied papers^2^). A full list of papers, their approach to screening, and a description of our categorisation procedure is available as Supplementary Material (SM1), whereas full descriptions of the tasks themselves can be found in SM2. We acknowledge that the resulting list of tests is surprisingly brief. Only one task (a variant of the CFMT paradigm, Tardif et al., 2019) failed to make our inclusion criteria because it had only been used on one occasion for super-recogniser screening, without any further uptake by the same or other labs. As few details were offered about this task, we have not included it in our review.

### Tests of face memory

Overall, the vast majority (80.00%; see Table 1 and SM1) of super-recogniser reports have used the extended form of the Cambridge Face Memory Test (CFMT+: Russell et al., 2009) for screening, with one also using an alternative version of the basic CFMT paradigm as a secondary measure (Tardif et al., 2019). This figure includes all theoretical investigations that have been published to date. Four papers from the applied literature did not use the task as they focused only on face matching (Davis et al., 2019; Noyes et al., 2018; Phillips et al., 2018; Robertson et al., 2016), and one additional paper sampled real-world “professionals,” using the CFMT+ post-inclusion to further investigate the skills of this group (Davis et al., 2018).

While the CFMT+ clearly dominates in terms of usage, two alternative unfamiliar face memory tests have been used much less frequently in super-recogniser screening: the Models Memory Test (MMT, Bate et al., 2018; Bate, Bennetts et al., 2020; Bate, Frowd, et al., 2019; a test which has recently gained traction with other labs and offers good reliability: Fysh et al., 2020) and the Adult Face Recognition Test (AFRT, Belanova et al., 2018; Robertson et al., 2020), which have only been used within the originating lab with unreported reliability. The Before They Were Famous (BTWF) test was used in the original report of Russell and colleagues (2009), but has only been adopted by one subsequent paper (Tardif et al., 2019). Notably, the University of New South Wales (UNSW) Face Test (Dunn et al., 2020) has very recently been published, offering some adequate psychometric properties for super-recogniser screening: test–retest reliability was reported as *r* = .59, and convergent validity with the CFMT+ was *r* = .31.

### Tests of face perception

Examination of Table 2 clearly demonstrates that (a) face perception tests are used much less frequently than face memory tests in super-recogniser screening (11 out of 25 papers used a perceptual test), and (b) when they are used there is no one task that is more popular than others. In part, this is because the two dominant tests that are used with the typical population (the Cambridge Face Perception Test, CFPT: Duchaine et al., 2007; and the Glasgow Face Matching Test, GFMT: Burton et al., 2010) have ceiling effects and are not appropriately calibrated for super-recogniser screening (see SM2). The remaining task, the Pairs Matching Test (PMT; Bate et al., 2018), has appropriate norms but has not yet appeared in papers authored outside of the lab where it was developed. There are also clear differences in the paradigms employed in face perception tasks depending on their motivation: those that originate from the cognitive neuropsychological literature are developed in line with more traditional measures that have been used to assess face perception skills in prosopagnosia (e.g., the CFPT), whereas tests from the forensic face matching literature aim to replicate more real-world tasks that require the comparison of two facial images (e.g., the GFMT and PMT).

Alternative face matching tasks have been used to identify individual differences in the typical population, and while they have potential for super-recogniser screening, they have not yet been used. For instance, participant accuracy on the Kent Face Matching Test (Fysh & Bindemann, 2018) is typically lower (66%–70%) than that attained on the GFMT, and the task has good test–retest reliability (*r* = .68 and .79 for match and mismatch trials, respectively). Stacchi et al. (2020) provide normative data for the Year Book Test (YBT; Bruck et al., 1991), which uses a simultaneous matching-to-array format for unfamiliar faces that differ substantially in age. A 6.7 SD difference was found between the control mean and ceiling, but task reliability was not reported. Fysh et al. (2020) trialled a short-version of the task (the YBT-10) for screening expediency, using only the 10 most difficult trials from the full version. They found that 3.16 SDs could be cleared between the control mean and ceiling, but the task only returned adequate levels of test–retest reliability (*r* = .44) and split-half reliability (α = .45–.62).

Finally, other matching tasks that have been used to further probe the skills of previously identified super-recognisers may be useful for screening itself, but inconsistently clear 2 SDs from the control mean before ceiling (e.g., the Models Face Matching Task, Dowsett & Burton, 2015; 1-in-10 Test, Bruce et al., 1999). Given the 2 SD cut-off is a somewhat arbitrary protocol, a higher clearance value may be desirable to ensure that screening tasks have sufficient sensitivity to discriminate between different grades of top-performers, for both philosophical and practical reasons.

## Screening protocols

Having reviewed the available super-recogniser screening tests, it is clear that there is some variability in the tasks used across laboratories, with the exception of consistent administration of the CFMT+. We now turn to the protocols involved in the administration of these tests, to identify (a) the specific combinations of tests that should be administered, (b) where the field lacks resources, and (c) precisely *how* screening should proceed.

*Which face memory tests should be administered?* The prosopagnosia literature gives some precedent for super-recogniser screening, and it seems particularly reasonable to follow these protocols when the motivation for many theoretical studies is to examine face recognition skills across the spectrum (Bobak et al., 2017; Russell et al., 2012; Tardif et al., 2019). While there is also considerable variation in the tests and protocols used for prosopagnosia screening (Bate, Bennetts, Gregory, et al., 2019; Robotham & Starrfelt, 2018), the possibility that the condition does not always present with deficits in face perception (Barton & Corrow, 2016; Dalrymple & Palermo, 2016) has focused diagnosis on tests of face memory.

Furthermore, it is typically recommended that prosopagnosia diagnosis should follow atypical performance on more than one face memory task (Barton & Corrow, 2016; Dalrymple & Palermo, 2016), overcoming task-specific issues with reliability, practice effects, borderline scores, and the “chance that it happened by chance” (Young et al., 1993, p. 945). Indeed, any person may perform within a range of scores that span several points surrounding their “true” ability, and several studies have demonstrated inconsistent performance across the same or similar tests in individuals within the typical population (Bindemann et al., 2012; Russ et al., 2018), those with developmental prosopagnosia (Murray & Bate, 2020), and super-recognisers (Bate et al., 2018; Bate, Frowd, et al., 2019; Bobak, Dowsett, & Bate, 2016). A more convincing case for categorisation into any of these participant groups would clearly be garnered from data that is collected across a battery of tasks, rather than reliance on a sole indicator.

The above principles of prosopagnosia screening can readily be applied to super-recognition. Here, there is already precedence towards using face memory tests during screening, with nearly all studies using the CFMT+ (see Table 1), and less than half of existing studies using a face perception measure (see Table 2). What is clearly missing is widespread use of more than one memory task at screening (see Table 3). Even if we eliminate from our 25 papers, the 2 that used “professional” experience as entry criteria for the specific aims of their study (Davis et al., 2018; Robertson et al., 2016), and 2 papers that amended their entry criteria to accommodate other aims of screening (Bate, Frowd, et al., 2019; Bobak, Pampoulov, & Bate, 2016), 12 of the remaining 21 papers only used one screening test (9 used the CFMT+ and 3 the GFMT; see SM1). Six papers required consistent performance on two tests (four used two memory tests, two used one memory and one perception test), and three papers used at least three tests (at least two face memory tests in each).

**Table 3.** Number of tasks administered during super-recogniser screening across all published papers to date.

No. of tasks	Process	Applied papers (*N* = 13)	Theoretical papers (*N* = 12)
0	Professional experience	2	0
1	Only face memory	4	5
1	Only face perception	3	0
2	Only face memory	1	3
2	Only face perception	0	0
2	Face memory and perception	2	2
3+	Only face memory	0	0
3+	Only face perception	0	0
3+	Face memory and perception	1	2

Thus, we recommend that, to keep consistency for theoretical comparison with the prosopagnosia literature, a minimum of two face memory tasks are administered (and show a demonstrable facilitation) in super-recogniser screening. Importantly, both tasks need to be appropriately reliable and we strongly recommend that one is the CFMT+. This allows direct comparison between super-recognisers, typical perceivers, and those with developmental prosopagnosia, both within- and between-studies (even where the short form of the CFMT has been administered to low-performing individuals). While there would be some advantages of specifying further common tests for use by all, there may also be benefits in the administration of different tasks by different laboratories, providing they have appropriate psychometric properties. This would not only overcome any practice effects that may result from multiple attempts at the same task (either by tests being made publicly available or because a participant has completed multiple screening batteries across different labs), but it also avoids any recommendations becoming overly prescriptive, allowing for personal preferences of researchers and the inclusion of new tasks. Finally, a test of famous face recognition is also acceptable when administered alongside standardised tests of unfamiliar face memory (Russell et al., 2009).

Finally, we also concur with the main practice in the prosopagnosia literature that an atypical score is one that falls at least 2 SDs from the control mean. There is already consistency in the use of the 2 SD cut-off in the more recent super-recogniser literature, with most papers adhering to this cut-off (e.g., Bate, Bennetts, Hasshim, et al., 2019; Bate et al., 2018; Bobak, Pampoulov, & Bate, 2016). For the CFMT+, as noted above, there has been a trend (Davis et al., 2018, 2020; Satchell et al., 2019) to use cut-off scores that are taken from pre-existing norming data (typically the cut-off of 90 taken from Bate et al., 2018; or 95/102 taken from Bobak, Pampoulov, & Bate, 2016). While this practice certainly makes sense for such a dominant task, it is important to note the difference in administration mode and demographics in the two samples. Bate et al. (2018) collected their data online of adults aged 18–50 years (*M* = 37.2), whereas Bobak, Pampoulov, and Bate (2016) collected their data face-to-face in a group of young adults aged 18–35 years (*M* = 21.4). These differences in sampling likely explain the difference in cut-off that was calculated in each study, indicating that consistency of participant age and testing modality are important factors in screening (see also Bennetts, Mole, & Bate, 2017).

Finally, it should be acknowledged that some papers have also included on-the-job performance or membership of a “professional” unit as super-recogniser inclusion criteria, either alongside objective verification (e.g., Davis et al., 2016, 2019) or on occasion, seemingly without (Davis et al., 2018; Robertson et al., 2016). Given that (a) researchers have not been able to disclose the screening protocols used by employing agencies, (b) there is a vast number of extraneous factors that may influence on-the-job face recognition performance (e.g., job role, familiarity with repeat offenders), and (c) there is little evidence to support the use of self-recommendations alone in super-recogniser screening (Bate & Dudfield, 2019; Bate et al., 2018; Bobak et al., 2017), we urge that the objective screening protocols recommended above are applied to all super-recogniser research participants, and these data are published regardless of professional status.

*What is the role of face perception tests?* The inclusion of face perception tasks in a super-recogniser testing battery is a more contentious issue. However, if we adhere to current understanding that super recognition (a) is primarily a facilitation in face memory, and (b) resides at the opposite end of a common face recognition spectrum to developmental prosopagnosia, then it follows that initial screening should focus on face memory tasks, without performance on follow-up tests of face perception influencing inclusion criteria. While this approach mirrors the prosopagnosia literature, admittedly it also in part reflects the absence of a gold standard face perception test, and the low diagnostic reliability that is associated with most existing face perception tasks (Bobak et al., 2017; Bobak, Pampoulov, & Bate, 2016).

Having said this, it is difficult to ignore claims of a single face recognition “factor” that reflects a more generalised face-processing ability covering both memory and perception (McCaffery et al., 2018; Verhallen et al., 2017): this more parsimonious hypothesis is certainly tempting for reasons of screening efficiency that would be better suited to real-world implementations of super-recognisers. Nevertheless, there is evidence to suggest that face perception is not facilitated in all super-recognisers (Bate et al., 2018; Bate, Frowd, et al., 2019; Davis et al., 2016; Robertson et al., 2020), although this conclusion is premature given the variation in screening protocols that have been reviewed above, with most studies relying on a single test and, in the case of face perception, those that lack appropriate calibration for top-performers. The same reservations apply to findings that a small number of super-recognisers have facilitations that are restricted only to face matching (Bate et al., 2018; Bate, Frowd, et al., 2019; Bobak, Hancock, & Bate, 2016), given thorough testing has not been performed. In fact, the dissociation between super-recognisers and “super-matchers” implies that there is not a common stage of facilitation that can be tapped at screening, as is the case for the two subtypes of prosopagnosia (all individuals are impaired at face memory, but only some at face perception).

It is at this point that we find theoretical investigations become most overwhelmingly at odds with more applied studies. In the former, it certainly makes sense for screening protocols to reflect those used for prosopagnosia screening. Yet, the vast majority of applied investigations have the ultimate aim of testing the abilities of super-recognisers for real-world identity matching tasks—fundamentally those that only involve face perception. Here, it can reasonably be argued that there is little to gain from the administration of face memory tasks, particularly if they do not always identify the same leading individuals as perceptual tasks and may even “miss” some “super-matchers” (e.g., Bate et al., 2018; Bate, Frowd, et al., 2019). However, this adjustment in protocol would lead the field away from a common definition of super-recognition, and we also argue that the limitations in screening protocols and the psychometric properties of perceptual tasks undermine existing work and make such a division premature. Instead, we recommend that studies primarily interested in face matching adhere to the protocol of administering at least two screening tasks, and using cut-offs that are 2 SDs from an appropriate control mean. If both tasks are perceptual in nature, follow-up testing should still report CFMT+ scores and performance on a supplementary face memory measure, to allow meaningful comparison across papers and to add rich data that can be consolidated across all studies to answer fundamental questions about the nature of super recognition.

*How should tests be administered?* Writing this article during a global pandemic, it is clear that the pre-2020 movement towards online psychological testing is here to stay. This is of course advantageous for purposes such as super-recogniser screening, where vast numbers of people from all geographical areas contact researchers on a daily basis in the belief that they have excellent face recognition skills. Given computerised face recognition tests are relatively easy to administer online, this mode of administration is also more time and cost efficient in terms of both participant travel and researcher time.

Nevertheless, there are issues associated with online testing that need to be carefully considered. Recent years have seen the advent of not only vast online participant recruitment banks, but also online testing platforms that are specialised for the administration of visuocognitive tasks, over and above surveys or questionnaires. Given these platforms ensure uniform screen size and presentation times, and capture accuracy and response time measures, it is prudent to use this technology. Issues of participant debrief and interpretation of performance nevertheless do need to be carefully considered. Researchers should enquire whether participants have taken part in previous screening studies, and ask them to share their scores rather than complete the same tasks again. For this reason, debriefs need to clearly advise participants of their scores and the names of the tests that they participated in, and ask them to keep this information on record should they seek participation elsewhere. This will assist with the issues of practice-effects and motivation, as considered above.

The same protocols should be applied to control participants. Given the public availability of the CFMT+ and its use in numerous studies worldwide, participants should be asked if they have previously completed the task, and excluded if that is the case. Existing norming data can be used where appropriate, but should match the age and ethnicity of the experimental group. If recruited from a participant recruitment website, a particularly large number of individuals will likely be needed, and tests should contain attention checks with data carefully monitored for signs of attention lapses and response bias (Buhrmester et al., 2018; Zhou et al., 2016). Ultimately, this does not solve the issue of participant incentive or motivation, and future research should carefully consider how an appropriate control sample can be identified and tested.

## Towards a definition and diagnostic protocols

Defining super-recognition is not easy, because it is very difficult to objectively tap the three behavioural characteristics of super-recognition that are identified at the start of this article. Thus, our definition of super-recognition is wholly constrained by the screening tests that we use to identify top-performers. This procedure is of course at odds with the fact that people self-refer for screening based on their experiences with faces in the real world—encounters that always have social and contextual meaning, even for people we have only just met. These circumstances are simply not replicated by the tasks of unfamiliar face recognition that are typically used to assess general face recognition ability. Rather, these tests typically present faces that have been cropped at least below the chin, and offer no contextual or semantic information about the person. Furthermore, we rarely encounter instances where we need to memorise or match completely unfamiliar faces in everyday life, unless employed in a relevant forensic or security occupation. Even if this is the case, most employees would never know their true error rate in these real-world tasks, given the ground truth is mostly untold. These considerations alone make it unsurprising that most people who self-refer for super-recogniser screening do not meet typical inclusion criteria that are derived only from performance on objective tests of unfamiliar face recognition (Bate & Dudfield, 2019; Bate et al., 2018), and raise further questions about whether laboratory-identified super-recognisers are truly those who excel at face recognition in the real-world.

While the same issue is true for the definition of prosopagnosia, difficulties in the real-world recognition of highly familiar faces tend to be more striking, given most people find this task exceptionally easy (Young & Burton, 2017). This characterisation feeds almost directly into common definitions of the condition: a profound and relatively specific difficulty in recognising the facial identity of even the closest family and friends (Barton & Corrow, 2016). If we take the same line of approach for a definition of super-recognition, reflecting on task difficulty, it follows that the definition should focus on the extraordinary ability of super-recognisers to readily perform what is arguably the most difficult face-processing task: recognising, from memory, unfamiliar faces that have only briefly been seen before. In this case, the definition actually complies with key screening tests, given our recommendation above to use multiple unfamiliar face memory tests as the dominant means to identify super-recognisers. To state this definition plainly, *super-recognisers are people who find it extraordinarily easy to recognise unfamiliar faces that they have only briefly seen before*.

Whether this definition can be extended to include face perception remains to be seen, once adequate screening tasks have been developed and large-scale data collection completed. However, if the skills of most super-recognisers do extend to face perception as current data suggests, this basic definition does not become redundant given it reflects the everyday real-world experiences of super-recognisers (as per the behavioural characteristics offered by Russell et al., 2009—these traits focus on memory rather than perception), and the dominant laboratory tests that are currently used to identify them. In this way, the definition is not intended to be restrictive or narrow, or even permanent, but to marry available data with the everyday experiences reported by super-recognisers. Likely, it will evolve in line with understanding. Furthermore, should more convincing evidence emerge for the existence of “super-matchers” (i.e., people who only have a superior ability to perceive faces, and not to remember them), then a separate definition would be useful. This is not unlike the prosopagnosia literature, where variations on the term have been offered to account for more specific patterns of performance that are of distinct theoretical interest (i.e., prosopamnesia, progressive prosopagnosia, or even associative versus apperceptive prosopagnosia; De Renzi et al., 1991).

Finally, a pertinent question concerns whether the protocols and definition offered above are adequate for real-world use of super-recognisers. While we have almost exclusively (and purposely) focused on the academic literature in this article, there is an increasing awareness that real-world forensic face recognition tasks are varied and influenced by multiple extrinsic and intrinsic factors (e.g., Fysh & Bindemann, 2017; Rumschik et al., 2020). As such, it is possible that (a) a person who performs highly on the inclusion tasks specified above does not have the additional qualities required to implement transfer of those skills to busy, often high-pressured, real-world occupational contexts, and (b) a generic face recognition factor either does not exist, or does not extend to every real-world context. The current consensus therefore seems to be that recruitment for real-world tasks should follow specific screening protocols that reflect the requirements of the task in hand.

Indeed, it is imperative to note that if screening for these applied roles had developed prior to, or at least independently of, the theoretical academic literature reviewed above, rather different tests would have been created and employed than those that have been used to date. Instead, the more applied avenue of super-recogniser screening has blindly followed the path that had already been set, without regard to relevant operational details that may impact performance in the real-world (e.g., task environment, time allowances, the availability of particular technology or tools, and the baseline ratio of target-present to target-absent trials). Clearly, attention to these additional issues would move our definition away from the one offered above, and the optimal individuals that are identified for some roles may not fulfil the original description at all. This leads us to question whether the rather informal term “super-recogniser” is either appropriate or helpful for real-world forensic settings, and whether the same individuals that we study for theoretical reasons are truly those that should be deployed in the real-world. Critically though, these issues cannot be resolved until we have a full battery of reliable, appropriately calibrated tasks that tap both face memory and face perception.

## Supplemental Material

sj-docx-1-qjp-10.1177_17470218211027695 – Supplemental material for When two fields collide: Identifying “super-recognisers” for neuropsychological and forensic face recognition research Click here for additional data file. Supplemental material, sj-docx-1-qjp-10.1177_17470218211027695 for When two fields collide: Identifying “super-recognisers” for neuropsychological and forensic face recognition research by Sarah Bate, Emma Portch and Natalie Mestry in Quarterly Journal of Experimental Psychology

## Notes

1. Here we include peer-reviewed empirical papers, and exclude opinion papers and reviews, conference proceedings, and pre-prints.

2. Papers were primarily allocated to each category according to journal. “Theoretical” papers were published in *Cognitive Neuropsychology, Cortex, iPerception, Journal of Exper-imental Psychology: Human Perception and Performance, Journal of Research in Personality, Neuropsychologia, Quarterly Journal of Experimental Psychology, Psychonomic Bulletin and Review*, and *Psychological Science*. Applied papers were published in *Applied Cognitive Psychology, Cognitive Research: Principles and Implications*, and *Forensic Science International*. Five papers were published in interdisciplinary journals and allocation was based on the motivation of the study, as set out in the title and aims (see SM1). This categorisation does not imply that applied papers cannot also have theoretical implications, but is merely a means to distinguish basic approach.
