# Supplementary material for: When two fields collide: Identifying “super-recognisers” for neuropsychological and forensic face recognition research
Source: Q J Exp Psychol (Hove). 2021 Jun 23;74(12):2154–64. doi: 10.1177/17470218211027695 (PMC8531948; doi:10.1177/17470218211027695)
Supplement: sj-docx-1-qjp-10.1177_17470218211027695 – Supplemental material for When two fields collide: Identifying “super-recognisers” for neuropsychological and forensic face recognition research [file sj-docx-1-qjp-10.1177_17470218211027695.docx]

Supplementary Material for:

**When Two Fields Collide:**

**Identifying “Super-Recognisers” for Neuropsychological and**

**Forensic Face Recognition Research**

Sarah Bate, Emma Portch and Natalie Mestry

**SM1:** **Published Papers Reporting Super-Recogniser Screening Protocols**

All papers that were identified via our literature search, exhausting all variations of the terms “super recogniser”, “super recognizer” and “super recognition”. Papers that did not include empirical data were excluded, as were published conference proceedings and pre-prints that had not been accepted for publication. The table indicates our classification of each paper into the applied (journals: *PeerJ; Cognitive Research: Principles and Implications; Applied Cognitive Psychology; PLOS One; Forensic Science International; Proceedings of the National Academy of Sciences of the USA*) or theoretical (journals: *Journal of Experimental Psychology: Human Perception and Performance; iPerception; Cortex; Cognitive Neuropsychology; Quarterly Journal of Experimental Psychology; Frontiers in Psychology; Neuropsychologia; Psychonomic Bulletin and Review; Journal of Research in Personality; Psychological Science*) literatures, primarily by journal, with interdisciplinary journals clarified by paper titles and aims. The screening tests and/or use of professional experience as inclusion criteria are summarised for each paper. Table inclusion focused on tests that had been unequivocally used for screening (i.e. cut-off scores and/or clearance above the control sample mean had been specified), rather than confirmation of group membership or as part of the empirical attempt of the paper.

| Paper | Field (words from title that justify classification where journal is interdisciplinary) | | Professional experience as entry criteria | Tests of Face Memory  (cut-off in SDs from control mean) | | | | Tests of Face Matching (cut-off in SDs from control mean) | | | Inclusion Criteria or recommended criteria (in terms of number of tests administered) |
| --- | --- | --- | --- | --- | --- | --- | --- | --- | --- | --- | --- |
|  | Journal | Classification |  | CFMT+ | MMT | BTWF | AFRT | CFPT | GFMT | PMT |  |
| Professional experience as initial entry criteria for further investigation | | | | | | | | | | | |
| Robertson et al. (2016) | Plos One (“police”) | Applied | Yes |  |  |  |  |  | Used post-inclusion |  | Unable to disclose professional screening protocols |
| Davis et al. (2018) | Applied Cognitive Psychology | Applied | Yes | Used post-inclusion |  |  |  |  |  |  | Unable to disclose professional screening protocols. Suspect identification rates considered for both police ‘experts’ and ‘identifiers’ |
| One test: CFMT+ | | | | | | | | | | | |
| Bennetts et al. (2017) | Cognitive Neuropsychology | Theoretical |  | 2 SDs |  |  |  |  |  |  | 1/1 |
| Bobak et al. (2016a) | Cortex | Theoretical |  | 1.84 SDs |  |  |  |  |  |  | 1/1 |
| Bobak et al. (2016b) | Plos One (“border control”) | Applied |  | 2 SDs |  |  |  |  |  |  | 1/1 |
| Bobak et al. (2016c) | Applied Cognitive Psychology | Applied |  | 1.4 SDs |  |  |  |  |  |  | 1/1 |
| Bobak et al. (2016d) | Frontiers in Psychology (“young adults”; aim was to examine inclusion criteria for theoretical work) | Theoretical |  | 2 SDs |  |  |  | Used post-inclusion |  |  | 1/1 (CFMT+ only); CFPT not recommended for future work |
| Bobak et al. (2019) | Quarterly Journal of Experimental Psychology | Theoretical |  | Not specified |  |  |  |  |  |  | 1/1 |
| Bobak et al. (2017) | Quarterly Journal of Experimental Psychology | Theoretical |  | 2 SDs |  |  |  |  |  |  | 1/1 |
| Davis et al. (2020) | Applied Cognitive Psychology | Applied |  | 2 SDs |  |  |  |  | Used post-inclusion |  | 1/1 |
| Davis et al. (2016) | Applied Cognitive Psychology | Applied |  | 1.94 SDs |  |  |  |  |  |  | 1/1 |
| One test: GFMT | | | | | | | | | | | |
| Davis et al. (2019) | Forensic Science International | Applied | Yes |  |  |  |  |  | 92.5% correct on short form (0.46 SDs) |  | 1/1 |
| Noyes et al. (2018) | Cognitive Research: Principles and Implications | Applied |  |  |  |  |  |  | 95% correct on short form (0.56 SDs) |  | 1/1 |
| Phillips et al. (2018) | Proceedings of the National Academy of Sciences of the USA (“forensic examiners”) | Applied | Some included on professional experience alone |  |  |  |  |  | 90% correct on long form (0.84 SDs) |  | 1/1 or professional experience |
| Two tests: CFMT+ and other memory test | | | | | | | | | | | |
| Bate et al. (2019b) | Journal of Experimental Psychology: Human Perception and Performance | Theoretical |  | 1.96 SDs | 1.96 SDs |  |  |  |  |  | 2/2 |
| Bate et al. (2020a) | iPerception | Theoretical |  | 1.96 SDs | 1.96 SDs |  |  |  |  |  | 2/2 |
| Belanova et al. (2018) | Cortex | Theoretical |  | 93/102 |  |  | Within 2 SDs of SR mean |  |  |  | 2/2 (Exps 1&2), 1/1 (CFMT+ only in Exp 3) |
| Robertson et al. (2020) | Applied Cognitive Psychology | Applied |  | 95/102 |  |  | Equalled or surpassed SR mean |  |  |  | 2/2 (Exp 3) |
| Two tests: CFMT+ and one other perception test | | | | | | | | | | | |
| Bate & Dudfield (2019) | PeerJ (“police”) | Applied |  | 1.96 SDs |  |  |  |  |  | 1.96 SDs | 2/2 |
| Bate et al. (2019d) | Applied Cognitive Psychology | Applied |  | 1.5 SDs |  |  |  |  |  | 1.5 SDs | 1/2; purposely liberal criteria for aims of study |
| Russell et al. (2012) | Neuropsychologia | Theoretical |  | Group comparison (SRs vs. controls) |  |  |  | Group comparison (SRs vs. controls) |  |  | 2/2 at the group level |
| Satchell et al. (2019) | Journal of Research in Personality | Theoretical |  | 95/102 |  |  |  |  | 100% accuracy |  | 1/2; analysed separably for each test |
| 3+ tests | | | | | | | | | | | |
| Bate et al. (2018) | Cognitive Research: Principles and Implications | Applied |  | 1.96 SDs | 1.96 SDs |  |  |  |  | 1.96 SDs | Index score |
| Russell et al. (2009) | Psychonomic Bulletin & Review | Theoretical |  | Group comparison (SRs vs. controls) |  | Group comparison (SRs vs. controls) |  | Group comparison (SRs vs. controls) |  |  | 3/3 at group-level |
| Tardif et al. (2019) | Psychological Science | Theoretical |  | 1.7 SDs; also 1.7 SDs on CFMT2 |  | Lowest SR score = 2.5 SDs |  | 1.7 SDs |  |  | 4/4 |

**List of abbreviations**

**AFRT:** the ‘Adult Face Recognition Test’ (Belanova et al., 2018)

**BTWF:** the ‘Before They Were Famous’ Test (Russell et al., 2009)

**CFMT+:** the ‘Cambridge Face Memory Test’ long form (Russell et al., 2009)

**CFPT:** the ‘Cambridge Face Perception Test’ (Duchaine et al., 2007)

**GFMT:** the ‘Glasgow Face Matching Test’ (Burton et al., 2010)

**MMT:** the ‘Models Memory Test’ (Bate et al., 2018)

**PMT:** the ‘Pairs Matching Test’ (Bate et al., 2018).

**SRs:** super-recognisers

***For paper references please refer to the exhaustive list in the main article.***

**SM2: Super-Recogniser Screening Tests**

**Tests of Face Memory**

***Cambridge Face Memory Test – Long Form (CFMT+; Russell et al., 2009):*** The CFMT+ is an extended version of the standard Cambridge Face Memory Test (CFMT; see Figure 1 in main article), a dominant test that is used worldwide to diagnose prosopagnosia (e.g. Bate & Tree, 2017; Barton & Corrow, 2016; Murray & Bate, 2020). In the CFMT, participants are required to learn the faces of six unfamiliar male individuals. They initially learn the identities one at a time, seeing each face from three different viewpoints, immediately followed by three test trials composed of triads of faces (a learned image of the target and two distractors). Participants then review the six targets simultaneously for 20 seconds, and then proceed to view 30 triads where they have an unlimited time to distinguish one target from two distractors. Here, targets are shown from novel viewpoints and/or lighting conditions than in the learning phase. After another 20-second review, participants complete a further 24 triads where targets are shown from novel viewpoints and with added visual noise. All faces are presented in greyscale and cropped to exclude external features. The standard version of the CFMT has been shown to have high internal reliability (α = .83, Herzmann et al., 2008; α = .88, Bowles et al., 2009), satisfactory test-retest reliability (*r* = 0.68; Murray & Bate, 2020) and both convergent and divergent validity (Bowles et al., 2009).

The CFMT+ is a more difficult test that is designed to be appropriately calibrated for the detection of super-recognisers (Russell et al., 2009). An additional 30 trials are added to the original CFMT paradigm, immediately proceeding the end of the task (see Figure 1). These images show faces from more extreme viewpoints and with different facial expressions, with additional visual noise. Some distractor identities are used repeatedly to make it harder to distinguish targets according to familiarity alone. Early work used group-based comparisons to distinguish super-recognisers from controls (Russell et al., 2009). However, an emerging single-case approach has been used in the subsequent literature. While the first studies simply matched their super-recogniser participants’ raw CFMT+ scores to those obtained by Russell et al.’s super-recognisers, these scores often fell short of the two SD from the control mean criterion (e.g. scores that were 1.4 SDs from the control mean were accepted in Bobak et al., 2016c, given they were equivalent to the raw scores obtained by Russell et al.’s super-recognisers). Later studies more consistently adhere to the two SD protocol, using either their own norming data (e.g. a raw score of 90/102 in Bate et al., 2018; or 95 in Bobak et al., 2016d) or that of others (e.g. Davis et al., 2018; Satchell et al., 2019; Belanova et al., 2018; Robertson et al., 2020; but see discussion in main article).

***Models Memory Test (MMT; Bate et al., 2018):*** While the CFMT+ uses tightly controlled facial images, the MMT was designed to embrace the more real-world natural variability that occurs between different presentations of the same face (Young & Burton, 2017), and to encompass target-absent trials. The procedure for learning six male identities and the use of triad test trials all follow the precedent of the CFMT+, but all images are shown in colour and are not cropped to exclude the external features. Following the learning phase and 20-second review, 90 test trials are presented, half containing a target and half without. A screen break is offered half way through the trials, but there is no further review of the target identities.

The inclusion of target-absent trials allows response bias to be examined, and separate scores on target-present and target-absent trials to be calculated. Performance on the CFMT+ correlated highly with target-present performance on the MMT, but less well with target-absent performance (Bate et al., 2018). Pertinently, only 37/89 individuals who were identified as super-recognisers using the CFMT+ also scored above cut-off (1.96SD above the control mean) on the MMT, raising questions about consistency of performance and the need for repeat-testing. More recent work has used the MMT in combination with the CFMT+ to further probe performance consistencies in super-recognisers (Bate et al., 2019d; Bate et al., 2020a). While this task appears in three papers from the originating lab, it now appears in external work (Fysh et al., 2020) and has been shared more widely. Recent reports demonstrate that between 3.14-3.34 SDs can be cleared between the control mean and ceiling (Bate et al., 2018; Fysh et al., 2020).

***Adult Face Recognition Test (AFRT; Belanova et al., 2018):*** The Adult Face Recognition Task utilises an old/new paradigm, sampling cropped unfamiliar faces. In an initial learning phase, participants view two blocks of 20 faces and, for each face, are given two seconds to decide whether the identity is older or younger than 30 years of age. Following a short break, participants begin the two recognition blocks, each of which contain an equal number of 20 new and 20 old faces, presented in a random order. For each face, participants are given an unlimited amount of time to decide whether the presented face is ‘old’ (previously seen during the learning phase) or ‘new’. Scores for both recognition blocks are combined to give a total score, out of 80.

The AFRT has been used as an additional screening measure to supplement the CFMT+. Rather a different approach to identifying super-recognisers was used in the two papers that have employed this test to date (from the same laboratory). In one paper, super-recogniser performance was required to be within two SDs from the super-recogniser mean for inclusion (Belanova et al., 2018), whereas Robertson et al. (2020) used a more conservative cut-off of 83% (i.e. the SR mean within their sample, prior to participant exclusions). Across both papers control means ranged between 75-80% correct, with SR performance at 0.8 (Belanova et al., 2018) and 1.4 (Robertson et al., 2020) SDs above the control mean. Using collapsed normative data from both investigations, the AFRT clears the control mean by 2.4 SDs before ceiling.

***Before They Were Famous Test (BTWF Test; Russell et al., 2009):*** As one of the hallmark symptoms of prosopagnosia is difficulties in familiar face recognition, famous face recognition tests are often used at screening (Barton & Corrow, 2016; Bate & Tree, 2017). However, these simplistic tests are not appropriately calibrated for super-recogniser screening because most people can complete them successfully. Instead, BTWF tests have been used to detect a broader range of individual differences in familiar face recognition skills. The original version of the task was used by Russell et al. (2009), who displayed 56 celebrity photographs that were captured prior to the onset of each individual’s fame, often during childhood. Participants view each face for three seconds, and then have unlimited time to name them or to provide a description that uniquely identifies each individual (e.g. a specific role they have played in a film rather than just “an actor”). No specific norming data has been presented for this task.

Familiar face recognition tests are advantageous in the sense that they more likely tap everyday face recognition experiences more closely than unfamiliar face recognition tests, and are more relevant to the super-recogniser characteristics identified by Russell et al. (2009; see paragraph 1 in the main paper). However, they are more laborious to implement, and norming data is likely not so accurate. Indeed, increased prior exposure to the target celebrities will inevitably lead to better performance regardless of underlying face recognition ability, and exposure varies widely between participants. Some attempt can be made to control for this possibility by removing any trials on a participant-by-participant basis where an individual reports low familiarity with the target celebrity, and modifying the proportion correct accordingly. In addition, this type of task is popular in online, magazine and ‘pub’ quizzes, and the scarce availability of BTWF images has led to repetition of content. Ensuring appropriate celebrities are selected for different participant groups is also important, as there are substantial age and nationality effects in celebrity face recognition. In fact, Russell et al. (2009) recommended that famous face tasks alone cannot identify super-recognisers (Russell et al., 2009), and only one further paper has used a BTWF test for super-recogniser screening (Tardif et al., 2019; although Robertson et al., 2016 used the ‘Pixelated Lookalikes Test’, which samples highly distorted familiar face images, in their investigation of pre-identified police super-recognisers). Tardif and colleagues reported that their super-recognisers performed at least 2.5 SDs above the control mean, with a specified cut-off of 1.7SD above the control means set for all other tasks.

**Tests of Face Perception**

***Cambridge Face Perception Test (CFPT; Duchaine et al., 2007):*** The CFPT is more commonly used to assess face perception skills in individuals with prosopagnosia, but it was also used in early investigations into super recognition (e.g. Russell et al., 2009, 2012). In this task, participants are required to sort six test images in order of their similarity to an unfamiliar target face. Each test image represents a ‘morph’ between the target and a discrete unfamiliar identity, with an incremental contribution of the latter until the test image represents only 28% of the target identity. There are eight upright and eight inverted trials, although both prosopagnosia and super-recogniser screening programmes tend to only consider upright performance (e.g. Bate & Tree, 2017; Russell et al., 2009; Tardif et al., 2019). Scores reflect the total number of errors made across all upright trials.

While the CFPT is sometimes used to tap individual differences in the typical population, its known psychometric properties indicate that it may not be suitable for the detection of super-recognisers. Performance on the task can be highly variable, and it is unclear whether this results from its complex instructions rather than genuine individual differences in face perception ability, particularly when administered online (Bowles et al., 2009; Bobak et al., 2016d). Further, the ratification adjustments applied to the facial images distance the paradigm from real-world forensic face matching challenges, and everyday face recognition experiences. Internal reliability of the upright face trials in the CFPT also varies widely (see Table 2), and Bobak et al. (2016d) found that the large variability in typical participants (and correspondingly large SD) incurred a ceiling effect, such that single-case comparisons on the performance of potential super-recognisers could not reach significance before ceiling. As such, it is unsurprising that the task has only been used in one further additional super-recogniser paper (Tardif et al., 2019).

***Glasgow Face Matching Test (GFMT; Burton et al., 2010):*** The GFMT was the first standardised face matching test to appear in the forensic face matching literature, motivated by an increasing awareness of the serious error-prone nature of face matching, even in experienced ‘professionals’ (e.g. passport control or police officers). It was well-received by the field, and remains a dominant task that is used to sample face matching skills in the typical population. The full version of the task includes 168 pairs of faces that sample both male and female identities, while a shorter version contains only the 40 most challenging pairs. In both versions of the task, faces are uncropped with half the trial pairs displaying faces of the same identity and the remainder showing faces of different identities. For ‘match’ trials, the two images of the same individual were captured minutes apart: one from a photographic camera, and the other a still image extracted from a video clip. In the task, participants make same or different identity judgements to simultaneously-presented faces under no time limit.

Here, we focus on the shorter task given it has most frequently been used for super-recogniser screening. This version demonstrates strong positive correlations between the first and last set of trials, indicating good internal reliability (*r* = .81; Burton et al., 2010). While Burton and colleagues found that scores ranged from 51-100% correct in 194 typical participants (M = 81.3%, SD = 9.7), other normative data suggests that average performance may be higher, with individual accuracy ranging from near-chance to perfect (Fysh & Bindemann, 2018; McCaffrey et al., 2018). Unfortunately, this calibration makes it unsuitable for super-recogniser screening, given two SDs cannot be cleared from the control mean before ceiling. Despite this, some super-recogniser studies have used the GFMT for screening, and even relied on this task as the sole entry criterion (Noyes et al., 2018; Phillips et al., 2018), or as a supplementary test to either the CFMT+ (Satchell et al., 2019) or “professional” screening (Robertson et al., 2016; Davis et al., 2019).

***Pairs Matching Test (PMT; Bate et al., 2018):*** This test has a similar design to the GFMT but was specifically calibrated for super-recogniser screening. In 48 trials, male (N = 24) or female pairs of faces are simultaneously presented for identity matching decisions (half match, half mis-match). Faces are presented in colour and are not cropped to exclude the external features. There is more variability between the matching identity images than in the GFMT, with photos often captured months apart. Participants make identity judgments in the same manner as the GFMT, without time limit.

Norms for the task allow 4.29 SDs to be cleared prior to ceiling, and the cut-off for super recognition is typically set at two SDs (Bate et al., 2018; Bate & Dudfield, 2019; a more liberal criteria of 1.5 SDs was used for different purposes in Bate et al., 2019d). In addition to these three investigations, the task was used for experimental purposes in two further papers (Bate et al., 2019b; Bate et al., 2020a), and is currently being used for super-recogniser screening by other labs. Task reliability was calculated by Bate et al. (2019b) as α = .74 for target-present trials, and α = .79 for target-absent trials.
